# Characterization of major ripening events during softening in grape: turgor, sugar accumulation, abscisic acid metabolism, colour development, and their relationship with growth

**DOI:** 10.1093/jxb/erv483

**Published:** 2015-11-17

**Authors:** Simone D. Castellarin, Gregory A. Gambetta, Hiroshi Wada, Mark N. Krasnow, Grant R. Cramer, Enrico Peterlunger, Kenneth A. Shackel, Mark A. Matthews

**Affiliations:** ^1^Wine Research Centre, the University of British Columbia, 2205 East Mall, Vancouver, BC V6T1Z4, Canada; ^2^Dipartimento di Scienze Agrarie ed Ambientali, University of Udine, 33100 Udine, Italy; ^3^Institut des Sciences de la Vigne et du Vin (ISVV), 210 Chemin de Leysotte, CS 50008, 33882 Villenave D’Ornon, France; ^4^National Agriculture and Food Research Organization, Kyushu Okinawa Agricultural Research Center, 496 Izumi, Chikugo, Fukuoka 833-0041, Japan; ^5^Department of Viticulture and Enology, University of California at Davis, 1 Shields Avenue, Davis, CA 95616, USA; ^6^Department of Biochemistry and Molecular Biology, University of Nevada, 1664 North Virginia Street, Reno, NV 89557, USA; ^7^Dipartimento di Scienze Agrarie ed Ambientali, University of Udine, 33100 Udine, Italy; ^8^Department of Plant Sciences, University of California at Davis, 1 Shields Avenue, Davis, CA 95616, USA

**Keywords:** Anthocyanins, cell wall, elasticity, firmness, fruit development, *Vitis vinifera* L.

## Abstract

Along with sugar accumulation and colour development, softening is an important physiological change during the onset of ripening in fruits. In this work, we investigated the relationships among major events during softening in grape (*Vitis vinifera* L.) by quantifying elasticity in individual berries. In addition, we delayed softening and inhibited sugar accumulation using a mechanical growth-preventing treatment in order to identify processes that are sugar and/or growth dependent. Ripening processes commenced on various days after anthesis, but always at similarly low elasticity and turgor. Much of the softening occurred in the absence of other changes in berry physiology investigated here. Several genes encoding key cell wall-modifying enzymes were not up-regulated until softening was largely completed, suggesting softening may result primarily from decreases in turgor. Similarly, there was no decrease in solute potential, increase in sugar concentration, or colour development until elasticity and turgor were near minimum values, and these processes were inhibited when berry growth was prevented. Increases in abscisic acid occurred early during softening and in the absence of significant expression of the *V. vinifera* 9-cis-epoxycarotenoid dioxygenases. However, these increases were coincident with decreases in the abscisic acid catabolite diphasic acid, indicating that initial increases in abscisic acid may result from decreases in catabolism and/or exogenous import. These data suggest that softening, decreases in turgor, and increases in abscisic acid represent some of the earliest events during the onset of ripening. Later, physical growth, further increases in abscisic acid, and the accumulation of sugar are integral for colour development.

## Highlight

The earliest events in ripening are decreases in turgor, softening, and increases in abscisic acid. Later events integral to regulating colour development include growth, further increases in abscisic acid, and sugar accumulation.

## Introduction

In fleshy fruits ripening is a complex event that encompasses many physiological and metabolic processes. Key compounds that determine fruit quality, such as sugars, pigments, and aromas, accumulate during this stage, and commonly fruits change their dimension and texture. Ripening has a strong impact on the final quality of the fruit and its economic value.

Grape (*Vitis vinifera* L.) berries are a non-climacteric fruit displaying a double sigmoidal growth curve in which two phases of rapid growth, stage I and III, are separated by a lag phase, stage II. The onset of ripening has been associated with the transition from stage II to stage III (Coombe, 1992) and involves softening, a decrease in acids, sugar accumulation, the resumption of growth, and colour development (in red varieties).

The onset of ripening is generally recognized to take place over a very short period of time and the conventional understanding is that berry softening is included among the processes commencing at this time (Coombe, 1992). Coombe and McCarthy (2000) write: ‘The second cycle begins with the onset of sugar accumulation, berry softening, berry colouring, and renewed size increase; taken collectively these events constitute veraison, denoting the beginning of ripening processes.’ However, recent studies examining the softening process in several grape varieties demonstrate that softening begins much earlier than previously recognized, during stage II, prior to rapid sugar accumulation, colour development, and the resumption of growth (Thomas *et al.*, 2008; Matthews *et al.*, 2009, Wada *et al.*, 2009).

In a variety of fruits, softening is largely attributed to the action of cell wall-modifying enzymes (reviewed in Brummell, 2006; Gapper *et al.*, 2013). In grape specifically, particular attention has been placed on expansins (Exp) (Ishimaru *et al.*, 2007; Schlosser *et al.*, 2008; Dal Santo *et al.*, 2013) as well as other enzymes, including pectin methylesterase (PME), pectate lyase (PL), and xyloglucan endotransglycosylase/hydrolase (XTH) (Barnavon *et al.*, 2000; Davies and Robinson, 2000; Yakushiji *et al.*, 2001; Nunan *et al.*, 2001; Schlosser *et al.*, 2008). In a recent study, 29 expansin genes were identified in the grapevine genome (Dal Santo *et al.*, 2013), but only a few members (including those investigated here) are expressed in the berry (Ishimaru *et al.*, 2007; Schlosser *et al.*, 2008; Dal Santo *et al.*, 2013). To date, no study has quantitatively related fruit firmness to the expression of these cell wall-related genes in grape berries.

While softening has been assumed to result from changes in cell wall structure, berry firmness is strongly associated with the turgor pressure (P) of mesocarp cells (Thomas *et al.*, 2008; Wada *et al.*, 2008; Matthews *et al.*, 2009). Similar observations of P decreases during softening in tomato (*Solanum esculentum*; Shackel *et al.*, 1991; Saladié *et al.*, 2007) and apple (*Malus* spp.; Tong *et al.*, 1999) indicate that decreases in P may serve as a primary mechanism of softening.

Previous studies by our laboratory demonstrated that the plant hormone abscisic acid (ABA) stimulates softening in cultured berries (Gambetta *et al.*, 2010), but the exact timing of changes in ABA metabolism in relation to softening and decreases in P is not known. ABA has a clear role in regulating ripening across diverse fruits (e.g. Koyama *et al.*, 2010; Gambetta *et al.*, 2010; Jia *et al.*, 2011; Zaharah *et al.*, 2012) and ABA is regularly applied in the field to stimulate ripening processes, and anthocyanin accumulation specifically (Cantín *et al.*, 2007; Peppi *et al.*, 2007; Buran *et al.*, 2012).

In grape, ABA concentrations increase and peak at the onset of ripening (Davies *et al.*, 1997; Owen *et al.*, 2009; Deluc *et al.*, 2009). There is evidence that increases in ABA result in-part through transient increases in the transcript abundance of the rate-limiting 9-cis-epoxycarotenoid dioxygenases (NCEDs) (Castellarin *et al.*, 2007; Wheeler *et al.*, 2009; Deluc *et al.*, 2009; Sun *et al.*, 2010). Active ABA can be inactivated in a variety of ways (reviewed in Finkelstein, 2013). It can be conjugated to glucose forming the inactive ABA-glucose ester (ABA-GE), which is stored or transported. Alternatively ABA can be catabolized by hydroxylation of at the 7′, 8′, or 9′ positions, although hydroxylation at the 8′ position produces phaseic acid (PA) and subsequently dihydrophaseic acid (DPA) predominates.

The goal of this study was to better characterize the physiological and molecular events during the softening of grape berries. In this work, softening is precisely quantified and expressed as elasticity (E) (Thomas *et al.*, 2008; Wada *et al.*, 2009). E represents the force–displacement relationship of a material, so a firm berry has higher E values (i.e. more pressure is required for a particular displacement), and as a berry softens E decreases. The measurement of E is non-destructive, allowing for the sampling of individual berries at precise moments during the softening process. In addition, we delayed E and P decreases and inhibited sugar accumulation by a mechanical growth-preventing treatment (Matthews *et al.*, 2009) in order to determine which processes are sugar and/or growth dependent. Here we examine the relationships between softening, decreases in turgor, changes in sugar accumulation, ABA metabolism, colour development, and the resumption of growth during the onset of ripening in grape.

## Materials and methods

### Plant material, treatments, and phenological measures

Grape berries (*V. vinifera* L. ‘Zinfandel’) were sampled from 20 field-grown vines located at the University of California, Davis, CA, USA (38.32′ N latitude and 121.46′ W longitude, elevation 18 m above sea level). The anthesis date was the day on which 50% of the cluster was flowering, and developmental time is represented as days after anthesis (DAA).

To analyse the evolution of berry E, 16 berries were randomly chosen and tagged 29 DAA, and the E of the intact berries was measured non-destructively at each sampling date with a custom fabricated instrument as described below.

For the main study, 80 clusters were randomly selected and tagged. To mechanically prevent the growth of developing berries, 195 boxes (length 17.5mm, internal diameter 10mm, thickness of the wall 1.0mm) were made with cryotubes (368632, NalgeNunc International, Rochester, NY, USA). The boxes were placed on individual berries (Box) at random within 48 randomly selected clusters (Supplementary Fig. S1) at 28 DAA (in stage I) between 11:00 and 15:00, when the berry diameter had reached 9.8mm on average, slightly smaller than the internal diameter of the boxes. Cryotubes were cut in half, the two walls were gently placed around the berries, and the boxes were secured with hose clamps (12684, NutsandBolts.com, Granby, MA, USA). The walls could be removed without damaging the berries using hose clamp pliers at later stages of development. The boxes were carefully removed from berries 79 DAA and these berries were left attached to the parent plant. A subset of berries were left boxed until the end of the experiment and used as boxed controls (Box Control). Mock boxes (Supplementary Fig. S1C) were designed to shield individual berries in a similar fashion as the boxes without restricting growth in an attempt to control for effects on berry microclimate. One mock box was placed on one berry of each of the 48 clusters. Visual assessment determined that berries shielded by mock boxes changed colour in a similar fashion to control berries, and there were no differences in decreases in E or increases in berry diameter and weight (Supplementary Table S1). Twenty to 100 (according to the berry developmental stage) control berries (Control) were randomly collected from the 80 clusters at 29, 41, 49, 52, 55, 58, 62, 69, 72, 76, 79, 80, 82, 85, and 90 DAA. Boxed berries (Box) were sampled at 29, 41, 49, 55, 58, 69, 79, 80, 82, 85, and 90 DAA. In general, berries were trimmed off the cluster at the pedicel and placed into zip-lock bags. Care was taken to avoid physical damage. To measure P, berries were immediately placed into aluminized Mylar zip-lock bags to prevent water and P loss (Thomas *et al.*, 2008). The bags were immediately placed into a Styrofoam box at ambient temperature and transported to the laboratory. When present, the boxes remained on the berry until berries were processed for measurements or stored at −80°C.

For each Control and Box berry, E was determined (described below). Individual Control berries harvested at 52, 58, 62, 69, 76, 79, 80, 85, 90 DAA were then used in subsequent analyses of both skin and flesh tissues on a per berry basis. For each sampling date, these further analyses were carried out on berries that spanned the range of elasticities present on that date, which varied over time (Supplementary Fig.S4A). The sample number used for each analysis is presented in the figure legends. All berry samples were stored at −80°C until these analyses were conducted. Box berries both before the box was removed (58, 69, and 79 DAA) and after the box was removed (80, 85, and 90 DAA) were compared with Control for all analyses. ABA analyses on Box berries were carried out only at 69 DAA and 85 DAA.

### Measurements of berry elasticity and turgor

At all sampling dates, berry E was measured as described in Wada *et al.* (2009). E was determined non-destructively using the GrapeGrabber, a custom fabricated instrument that measures force and displacement during berry compression. As described in Thomas *et al.* (2008), the observed force–displacement relationship was fitted using SAS PROC NLIN (version 8.2; SAS Institute) to the model expected for compression of a perfectly elastic sphere, or column in the case of boxed berries (Hertz equation; Wada *et al.*, 2009). Box berries were removed from the box before assessing E.

To measure P, Control berries were analysed at 41, 49, 55, 58, 62, 69, 79, 82, 85, and 90 DAA, Box berries at 41, 49, 55, 69, 79, 80, 82, 85, and 90 DAA, and Box Control berries at 79, 85, and 90 DAA. The cell pressure probe technique (Hüsken *et al.*, 1978), modified as described previously (Shackel *et al.*, 1987), was utilized to measure the P of individual cells in the berry mesocarp. For all treatments, measurements were taken between depths of 100 and 2500 µm from the epidermis, approximately 3mm away from the pedicel–berry junction of each berry, using a Piezo micromanipulator (PM-10, Stoelting Co., IL, USA). For Box berries, when necessary, a portion of the polyethylene box wall covering the berries was carefully pared with a razor blade to avoid interference with the field of view during microscopic observation and to ensure an adequate working distance to the berries (Thomas *et al.*, 2006). Microcapillary tips were prepared as described previously (Shackel *et al.*, 1987) to create 3–4 µm (outer diameter) tips at the widest point of the bevelled portion. All measurements were performed under laboratory conditions (diffuse fluorescent light, 25°C air temperature, and localized 100% relative humidity obtained with a humidifier) and were completed within 3h of detachment from the cluster. Previous work has shown that berry P does not significantly change for up to 48h after being excised from the vine if water loss from the berry is prevented (Thomas *et al.*, 2006). Reported *P*-values represent averages of 12–42 cells from two to six berries collected from independent plants, except for Box Control berries at 90 DAA (12 cells from one berry).

### Measurements of total soluble solids, solute potential, and sugar concentration

Subsets of harvested berries were used to determine total soluble solids. Sap was collected by squeezing six to nine Control and Box berries. Three berries were used for Box Control at 85 DAA and one at 90 DAA. Total soluble solids were measured with a handheld refractometer (Reichert A2R200, ReichertGmbH, Seefeld, Germany) and reported as °Brix.

To determine solute potential, berries were deseeded and peeled with a scalpel and placed in 2mL tubes. In order to avoid contamination of the skin with flesh sap, skins were gently blotted with a Kimwipe before storing in the tube. The tissue solute potentials from skin and flesh tissues were measured using the supernatant obtained by centrifuging tissues at 2000 × *g* for 10min after thawing at 25°C, as reported by Wada *et al.* (2008). An aliquot of 5 µL was used to determine tissue solute potentials (5500 Vapor Pressure Osmometer, Wescor Inc., Logan, UT, USA). Whole berry solute potential was calculated by averaging the contribution of the flesh and the skin for each measured berry.

Another supernatant aliquot of 5 µL was diluted 1:50. The concentration of glucose and fructose was quantified using an Agilent capillary electrophoresis system (G1600AX, Agilent Technologies, Germany) according to Soga and Imaizumi (2001) and Wada *et al.* (2008). In short, separations were carried out on fused silica capillaries preconditioned for 5min by flushing with Agilent basic anion buffer. The sample was injected with a pressure of 50 mbar for 6s and the applied voltage was set at −30kV, and the capillary thermostat set to 15°C. The detection wavelength was set at 350nm for the constant signal wavelength and at 230nm for the reference wavelength. Unknown peaks were identified using a co-electropherogram with internal standard solutes.

### Quantification of anthocyanins

Anthocyanins were extracted in 1/10 (w/v) skin/solvent suspension of 50% methanol in water for 20min in an Ultrasonic Cleaner bath (Downey *et al.*, 2007). After centrifugation at 13 000 × *g* for 15min, samples were injected into an Agilent 1100 Series HPLC system equipped with a SGE Wakosil II C18 RS column (3 µm, 150×4.6mm) and a guard SGE Wakosil II C18 RS cartridge. Gradient conditions were as reported by Bogs *et al.* (2005). Total anthocyanin content was expressed as milligrams of malvidin 3-glucoside equivalents per berry and included monoglucoside, acetyl-glucoside, and *p*-coumaroyl-glucoside fractions.

### Quantification of ABA and related metabolites

ABA extraction was based on a previously published method (Deluc *et al.*, 2009), with some modifications. An internal, deuterated standard mixture (IS mix) was prepared containing (+)- 4,5,8′,8′,8′-d5-ABA-GE and (-)-5,8′,8′,8′-d_4_ ABA (Plant Biotechnology Institute, Saskatoon, Canada). The non-labelled mixture included (+)-ABA-GE and (±)-7′OH-ABA and was used to produce quality control standards, which were prepared and analysed along with each batch of samples. Both mixtures were prepared in a water, acetonitrile, and glacial acetic acid mix (H_2_O:ACN:AcOH, 50:49.5:0.5) containing equal amounts of each standard (3ng μL^−1^). The reconstitution mix was dissolved in the starting solvent of the HPLC gradient (H_2_O:ACN, 85:15, with 0.1% AcOH).

Aliquots of 50–100mg of lyophilized whole grape berry samples were extracted in 3mL of isopropyl alcohol:H_2_O:AcOH (80:19:1) along with a 60ng equivalent of each of the deuterium-labelled standards. After vortexing for 1min, the solution was put onto an orbital shaker at ~4°C for 24 hours. After extraction, the samples were centrifuged for 20 minutes at 2500 × *g* and the supernatant removed and transferred to a glass test tube (2mL screw thread). The solids left in the centrifuge tube were washed with another 0.5mL portion of the extraction solvent, vortexed and centrifuged again, and the supernatant added to the first portion. The supernatant was dried in a centrifugal concentrator (GMI, Ramsey, MN, USA) at room temperature and 10 × *g*.

The dried extract was reconstituted in 2mL of H_2_O:AcOH (99:1) for clean-up by mixed-mode cation-exchange solid-phase extraction (Oasis MCX SPE cartridges, 3 cc, 60mg; Waters, Milford, MA, USA). The cartridges attached to a vacuum manifold were first washed with 3mL MeOH:AcOH (99:1) and then 3mL H_2_O:AcOH (99:1). Samples were then added to each cartridge and allowed to run through at a rate of approximately one drop every 1–2s. The sample vials were washed with 0.5mL H_2_O:AcOH (99:1) and this wash was added to the appropriate cartridge. Each cartridge was washed again with 1mL H_2_O:AcOH (99:1). The cartridges were eluted after centrifugation at 45 × *g* for 1min into 1.5mL of MeOH:AcOH (99:1) by adapting the cartridges onto a microcentrifuge. Finally, the eluate was dried in a centrifugal concentrator at room temperature.

The dried extracts were re-dissolved in 100 μL MeOH:AcOH (99:1) and made up to 1mL in H_2_O:AcOH (99:1) for a second stage of solid phase extraction using hydrophilic–lipophilic balance cartridges (Oasis HLB, 1 cc, 30mg; Waters). The cartridges were prepared by washing and equilibrating with 1mL MeOH:AcOH (99:1) and 1mL H_2_O:AcOH (99:1), respectively, before loading the sample. The drip rate was approximately one drop every 1–2s, and a second wash of 0.5mL H_2_O:AcOH (99:1) was added as the sample was drawn through the cartridge. The sample was washed with 1mL H_2_O:AcOH (99:1) and finally eluted into a second microcentrifuge tube using 1mL H_2_O:ACN:AcOH (69:30:1). This final extract was dried in a centrifugal concentrator (GMI) and re-dissolved in 80 μL of the reconstitution mix prior to LC-MS/MS analysis.

All samples were analysed using a Michrom Paradigm MDLC (Michrom Bioresources Inc., Auburn, CA, USA) coupled to an LCQ DECA XP+ ion trap mass spectrometer (Thermo Finnigan, San Jose, CA, USA). The flow rate was set to 200 μL min^−1^ using a Symmetry C18 column (2.1×100mm, 3.5 um; Waters) and auto-injection of 20 μL using a Paradigm AS1 (Michrom Bioresources Inc.). The electrospray mass spectrometer was operated under negative ion mode using a needle potential of 2.50kV with a capillary temperature of 220°C and a sheath flow of 20 psi.

### Gene expression analyses

Berries for RNA extraction were peeled with a scalpel while still frozen and the obtained tissues, skin, and flesh were stored at −80 °C till the RNA extraction. Total RNA was extracted from 0.1–0.3g of tissue following the procedure described by Iandolino *et al.* (2004) and treated with 0.5U µg^−1^ RQ1 DNase (Promega Corporation, Madison, WI, USA). First-strand cDNA was synthesised using ~0.5–2 µg of RNA, 0.5 µM (dT)18 primer, and 50U of M-MLV Reverse Transcriptase (Promega Corporation, Madison, WI, USA). Quantitative real-time reverse transcriptase PCR (qPCR) was carried out as described in Gambetta *et al.* (2013). The absolute expression level of *VviUbiquitin1* was determined from standard curves established from genomic DNA standards, and absolute levels of expression for each gene were determined. Gene expression was expressed as the copy number per nanogram of RNA as previously described (Gambetta *et al.*, 2013), with the mean from three biological replicates reported. The grapevine reference genome V1 annotation (Jaillon *et al.*, 2007) for the genes tested in this study as well as the references for the primer’s sequences are reported in Supplementary Table S2.

### Statistical analyses

In general, ANOVAs were carried out to compare all parameters used across time (DAA) or E ranges by each treatment, and between skin and flesh tissue. For treatment effects, means at each sample date were compared by Tukey’s honest significant difference (HSD) test. All expression data were log transformed according to Rieu and Powers (2009) prior to statistical analysis. All analyses were carried out using JMP-SAS software (SAS Institute Inc., http://www.sas.com). Linear and non-linear regressions were carried out using built-in curve models in the JMP-SAS software.

## Results

### Berry growth, soluble solids, and colour development

**Fig. 1.**
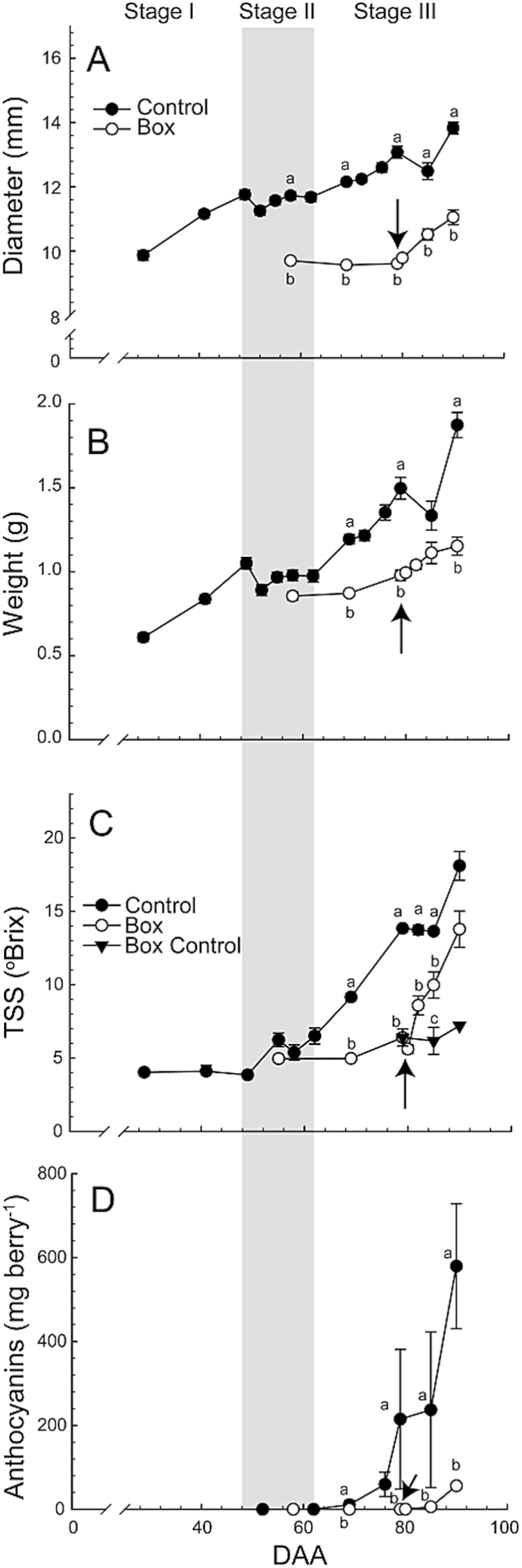
Berry diameter, weight, and total soluble solids (TSS). (**A, B**) Berry diameter and weight in Control and Box berries (n = 20–100). (**C**) Degrees Brix in Control (n = 6–9), Box (n = 6), and Box Control (n = 3 at 85 DAA and n = 1 at 90 DAA). (**D**) Anthocyanin accumulation in berry skins as a function of DAA in Control and Box (n = 3). Boxes were removed at 79 DAA (arrows) and Box Control remained boxed on the cluster. Bars represent ± SE and different letters signify significant differences (Tukey’s HSD, *P* < 0.05).

Control berries exhibited the typical double sigmoid growth habit, with more rapid increases in diameter and weight early in development (until ~48 DAA), and after 62 DAA (Fig. 1A, B). Growth was prevented in Box berries using modified cryotubes and tubing clamps (Supplementary Fig. S1). The boxes could be removed without harming the berry by simply removing the clamp. Boxes restricted berry diameter to the inner diameter of the box (Fig. 1A), but an approximate 10–15% increase in weight still occurred (Fig. 1B) associated with a lengthening of the berry (Supplementary Fig. S1B). Boxes were released from the berries at 79 DAA, and both berry diameter and weight began to increase within 24 hours (Fig. 1A, B). After the boxes were removed, berry weight increased to approximately 60% of Control berries by 90 DAA.

Preventing growth inhibited the accumulation of sugars in berries (Fig. 1C). Sugar accumulation in Control berries began rapidly at approximately 60 DAA, but Box berries did not accumulate sugars to any significant extent until after the boxes were removed at 79 DAA. After removing the boxes, these berries accumulated sugars significantly faster (Supplementary Fig. S2), and soluble solid levels were greater than 80% of Control berries by 90 DAA. Soluble solid level remained low in Box Control berries.

Control berry skins began to accumulate anthocyanins at 69 DAA (Fig. 1D), by which time E was less than 2 MPa (Supplementary Fig. S3A). Box berries did not develop colour if the box was left in place, indicating that anthocyanins did not accumulate until the boxes were removed at 79 DAA (Fig. 1D). Individual berries rapidly developed colour after boxes were removed: 40% of berries exhibited colour development in less than 48 hours (Supplementary Fig. S3B). Although anthocyanin concentration was still increasing at 90 DAA, levels were still only a fraction of Control values (Fig. 1D).

### Softening and turgor

**Fig. 2.**
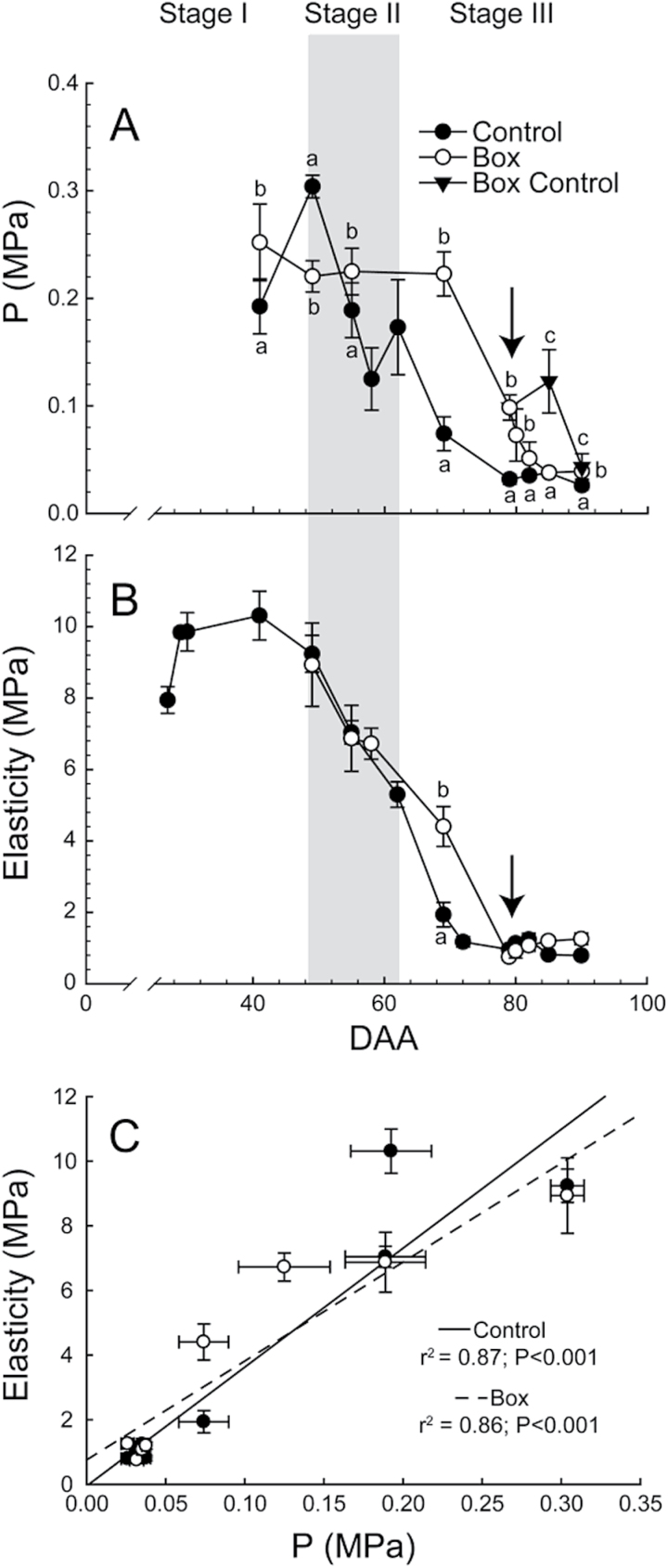
Cell turgor (P) and berry elasticity. (**A**) Cell turgor through berry development in Control, Box, and Box Control (n = 12–42). (**B**) Elasticity in Control and Box berries (n = 16). Boxes were removed at 79 DAA (arrow) and Box Control remained boxed on the cluster. Bars represent ± SE and different letters signify significant differences (Tukey’s HSD, *P* < 0.05). (**C**) Relationship between elasticity and cell turgor. Points are means of 2–6 berries and bars are ±SE.

Berry turgor (P) peaked in Control berries at the transition between stage I and stage II, then decreased strongly through the start of stage III, and remained stable or declined slightly afterwards (Fig. 2A). The E pattern was similar to P but the maximum E value was observed slightly earlier (Fig. 2B). E and P were strongly and significantly correlated with each other (Fig. 2C).

Box berries did not exhibit a peak in P at the transition between stage I and stage II (Fig. 2A). Preventing growth delayed, but did not completely inhibit, berry softening, see particularly Box at 69 and 79 DAA, and Box Control at 85 DAA. In Box berries, P decreased to Control levels less than a week after the boxes were removed. However, even in Box Control berries, P decreased to Control levels by 90 DAA (Fig. 2A).

In Box berries, E was comparable to Control at 58 DAA (Fig. 2B), and decreased thereafter, although more slowly than Control berries. Box berries had comparable E to Control berries even before the boxes were removed at 79 DAA.

The heterogeneity of berry E in the vineyard changed dramatically across fruit development (Supplementary Fig. S4A). The heterogeneity increased approaching the transition between stage II and stage III, then declined rapidly as berries reached low E. Colour development was visually observed only after berries reached an E below 2MPa (Supplementary Fig. S4A). Similarly, both berry weight and berry diameter did not appear to increase until approximately the same time; after berries reached an E below 2MPa (Supplementary Fig. S4B, C).

### Expression of key cell wall-modification enzymes in berry skin and flesh

**Fig. 3.**
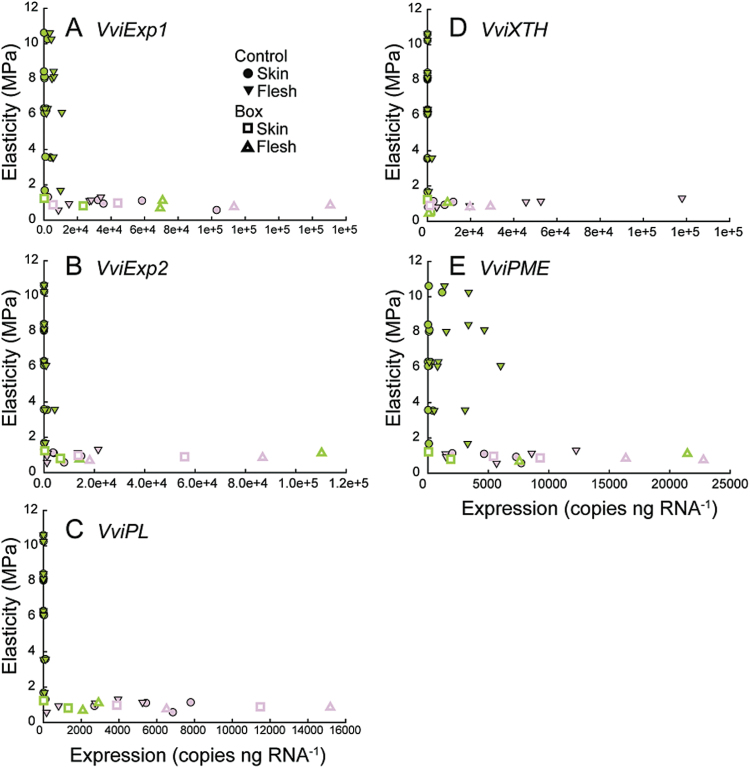
Expression of key cell wall metabolism enzymes in skin and flesh as a function of elasticity. Expression of *VviExp1* (**A**), *VviExp2* (**B**), *VviPL* (**C**), *VviXTH* (**D**), and *VviPME* (**E**) in skin and flesh as a function of the elasticity in Control and Box berries. Each point represents the level of expression in the skin or flesh of a single berry at the corresponding measured E. Points are coloured according to berry skin colour.

Changes in the expression of five genes thought to act in fruit softening were quantified in the flesh and in the skin of individual Control and Box berries, and plotted versus each berry’s E (Fig. 3). These genes included the two predominately expressed expansins (*VviExp1* and *VviExp2*) in grapevine berry, a pectate lyase (*VviPL*), a xyloglucan endotransglycosylase/hydrolase (*VviXTH*), and a pectin methlyesterase (*VviPME*). All these genes, except *VviEXP1* and *VviPME*, exhibited similar patterns of expression: the transcript abundance being low or completely absent as E decreased from ~10 to ~2MPa and relatively high in berries with lower E. In most cases these genes were not up-regulated until E was below 2MPa, and expression was higher in flesh tissues. *VviExp1* (Fig. 3A) and *VviPME* (Fig. 3E) were the only two genes that had significant levels of expression at high E, but again only in berry flesh. After colour development, expression of all the genes was up-regulated in skin and flesh (Fig. 3). In general, expression in Box berries was on par with Control berries (Fig.3).

### Berry solute potential and sugar accumulation

No significant differences were detected for the solute potential between the Control tissues, berry skin (Ψ_s_^Skin^), and flesh (Ψ_s_^Flesh^), independent of the DAA and the E of the berries considered (Supplementary Table S3). The solute potential of the Box berries at 58 DAA was significantly lower in the flesh than in the skin (Supplementary Table S4), but for the rest of the harvesting points, no significant differences were detected. Therefore, we present the Ψ_s_ of the berry (Ψ_s_^Berry^) as an average of the Ψ_s_ of the two tissues. When considering sugar concentration in berry skins and flesh separately, statistical analyses revealed significant differences between tissues only in Control berries before the large accumulation of sugars started, with flesh having higher sugar concentrations than skin (Supplementary Table S3). We present the glucose and fructose concentration of the berry (reported as millimolar) as an average of the concentrations of the two tissues for simplicity.

**Fig. 4.**
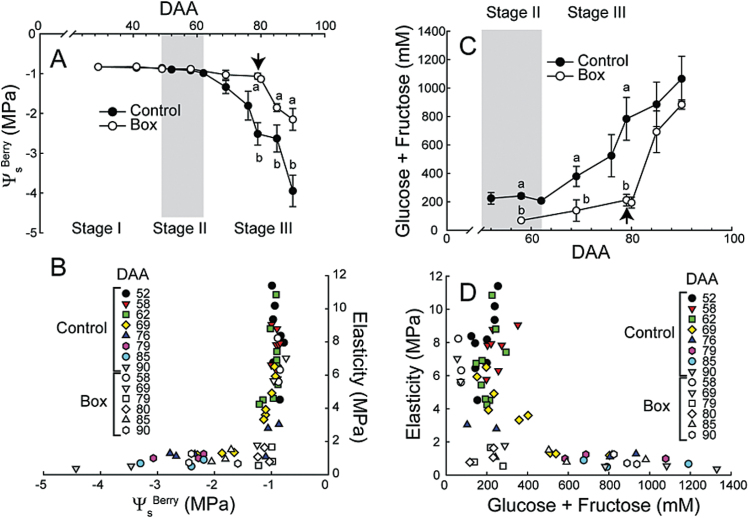
Relationships between Ψ_s_^Berry^, elasticity, and sugar concentration. (**A**) Ψ_s_^Berry^ in Control and Box berries. Boxes were removed at 79 DAA (arrow). Bars represent ±SE and different letters signify significant differences (n = 3–9 for Control and n = 3 for Box; Tukey’s HSD, *P* < 0.05). (**B**) The relationship of berry Ψ_s_^Berry^ to the elasticity of Control and Box berries. (**C**) Developmental changes in berry sugar concentration (glucose + fructose) in Control and Box berries. Boxes were removed at 79 DAA (arrow). Bars represent ±SE and different letters signify significant differences (n = 3–9 for Control and n = 3 for Box; Tukey’s HSD, *P* < 0.05). (**D**) The relationship between the elasticity and sugar concentration in Control and Box berries.

In Control, berry solute potential (Ψ_s_^Berry^) was constantly high until 62 DAA, decreased to −1.3MPa at 69 DAA, and decreased rapidly afterwards (Fig. 4A). In Box berries, preventing growth inhibited decreases in Ψ_s_^Berry^ until the boxes were removed (Fig. 4A). Significant decreases in Ψ_s_^Berry^ did not occur until E was below 2MPa (Fig. 4B). This relationship was independent of harvesting date (Fig. 4B).

Control berry sugar concentration was low until 62 DAA and then progressively increased. Box berries did not accumulate large amounts of sugar until the boxes were removed at 79 DAA; prior to the removal of the box, sugar concentrations were significantly lower than Control. After boxes were removed, sugar concentration rapidly increased and was nearly equal to Control levels at 85 and 90 DAA (Fig. 4C). Berry sugar concentration did not change significantly until E reached very low levels (< 2MPa) regardless of the DAA when berries were harvested. In Box berries, sugars remained low until boxes were removed even if berries had reached low levels of E (~1MPa) before that time (Fig. 4D).

### Expression of key sugar accumulation enzymes in berry skin and flesh

**Fig. 5.**
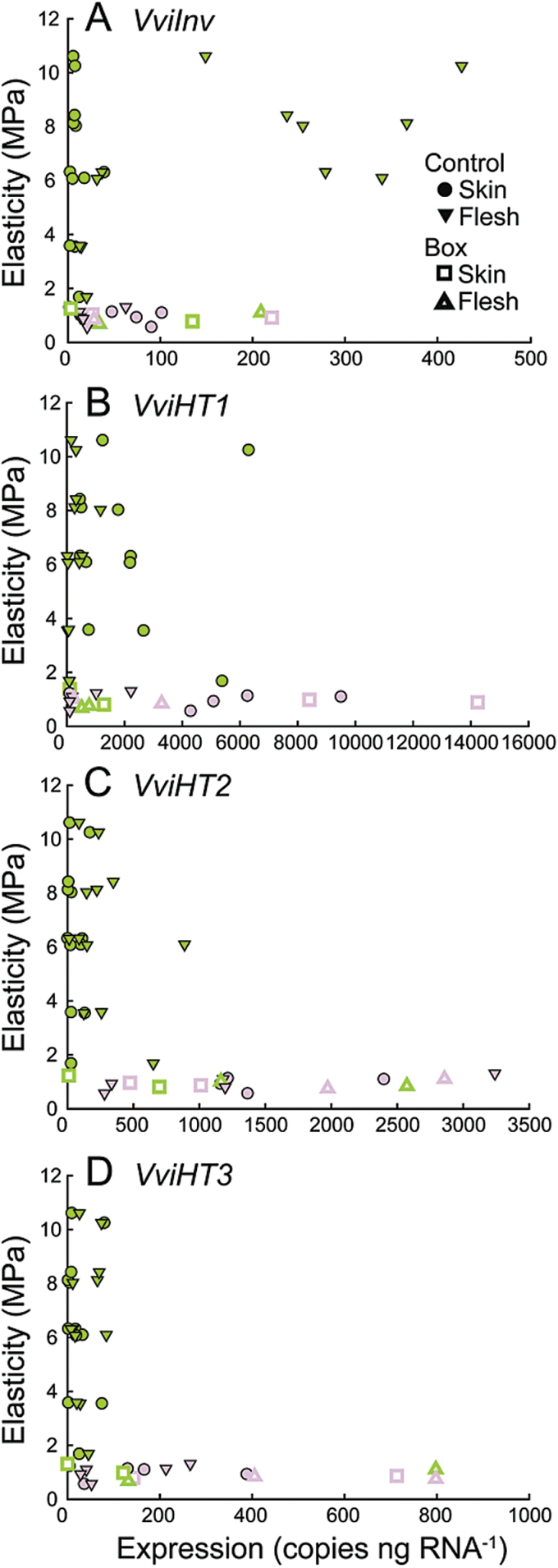
Expression of key sugar metabolism enzymes in skin and flesh as a function of elasticity. Expression of *VviINV* (**A**), *VviHT1* (**B**), *VviHT2* (**C**), and *VviHT3* (**D**) in skin and flesh as a function of the elasticity of Control and Box berries. Each point represents the level of expression in the skin or flesh of a single berry at the corresponding measured E. Points are coloured according to berry skin colour.

*VviInv* was highly expressed in berry flesh at high E (Fig. 5A). Expression decreased to low levels as E approached 2MPa. In contrast, *Vvilnv* expression in skins remained very low until E was below 2 MPa and then slightly increased.

*VviHT1* was expressed at much higher levels than *VviHT2 and VviHT3*, with peak expression levels being as much as four times greater than those of *VviHT2* (Fig. 5B)*. VviHT1* was predominantly expressed in berry skins. *VviHT1* expression varied as E decreased: at values below 2MPa, and particularly after colour development, expression increased in skins. In green berries at high E, *VviHT2* and *VviHT3* were predominantly expressed in flesh (Fig. 5C, D). At low E (E < 2MPa) and after colour development, expression of both genes increased in flesh and skins.

### Changes in ABA metabolism

**Fig. 6.**
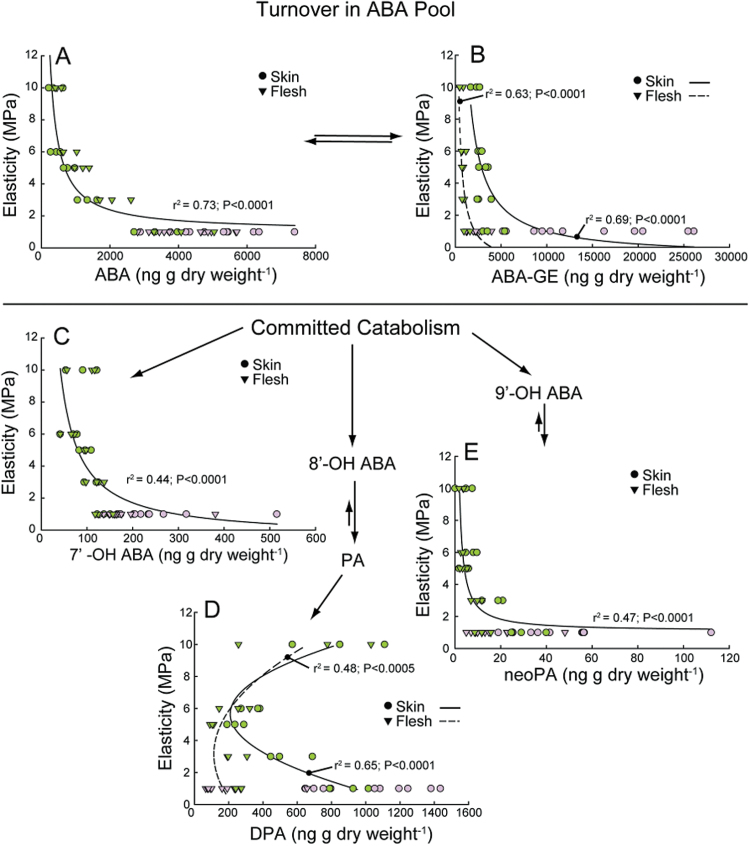
ABA metabolism. Concentration of ABA species, including active ABA (**A**), its inactive glucose ester conjugate ABA-GE (**B**), and the committed catabolites 7′-OH ABA (**C**), diphasic acid (**D**), and neophasic acid (**E**), as a function of elasticity. Box treatment data include both boxed and unboxed berries because treatment had no significant effect. Each point represents the level of metabolite in the skin or flesh a single berry. Points are coloured according to berry skin colour.

Active ABA, its inactive glucose ester ABA-GE, and four committed ABA catabolites were quantified in berry skins and flesh across various E (Fig. 6). Box berries were analysed at two time points; in green berries prior to box removal, and in red berries 1 week after the removal of the box. There were no significant differences (*P* > 0.05) in ABA, ABA-GE, or any catabolites between control and boxed/unboxed berries at these points in time so these data were pooled with control data for the following analyses.

ABA concentrations changed similarly in both skins and flesh, remaining low until E dropped to 5MPa, when ABA concentrations began to increase (Fig. 6A). As E decreased below 5MPa, ABA concentrations increased further. ABA concentrations reached near peak levels in green berries at low elasticity. ABA-GE was consistently higher in the skin than in the flesh tissues (Fig. 6B). At high E there was little difference in ABA-GE concentrations between skin and flesh, but as E decreased ABA-GE accumulated to a much greater extent in skins. This difference persisted after colour development.

7′-OH ABA and DPA were the predominant ABA catabolites present (Fig. 6C, D), and concentrations of 9′-OH ABA, known as neophasic acid (neoPA), were considerably lower (Fig. 6E). 7′-OH ABA and neoPA changed similarly with regard to E in both the skin and flesh. The concentrations of these metabolites were low at high E, remained relatively low during softening, and then increased substantially in coloured berries at low E (Fig. 6C, E) and high ABA concentrations. DPA, in contrast to ABA, ABA-GE, 7′-OH ABA, and neoPA, was detected at relatively high levels at high E in both skin and flesh (Fig. 6D). As E decreased, DPA concentrations initially decreased. However, DPA concentrations increased again in skin at low E, while concentrations remained low in the flesh.

### VviNCED *expression, ABA, sugars, and anthocyanins*

There were no significant differences in expression between *VviNCED1* and *VviNCED2*, by tissue type, development time (DAA), or E (*P* > 0.05). Equally, we found no significant differences in ABA concentrations between tissues when compared within development times (DAA) or at similar E (*P* > 0.05). Therefore, we present *VviNCED* expression and ABA concentration regardless of tissue.

**Fig. 7.**
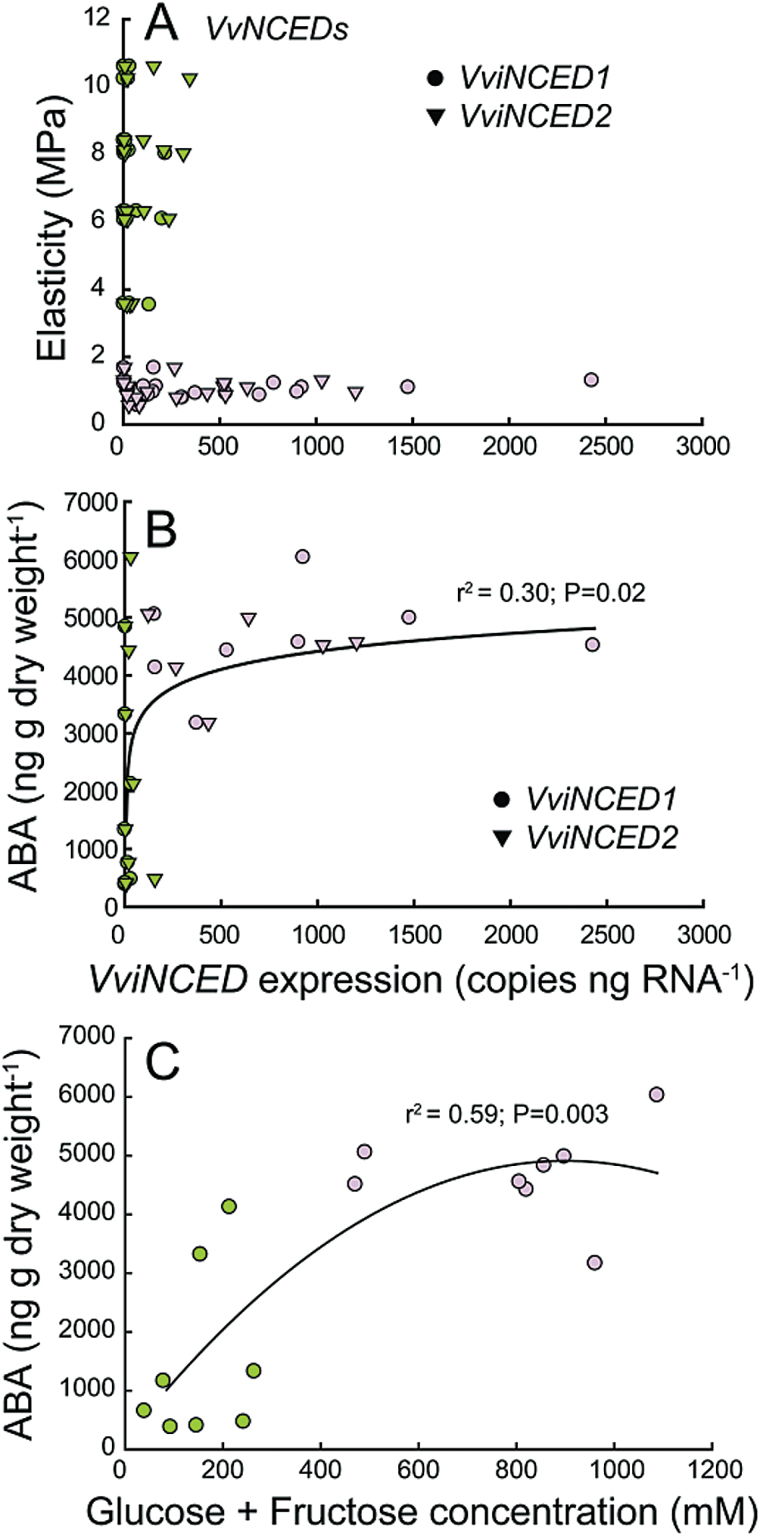
Relationships among *VviNCED* expression, ABA concentration, and sugars. (**A**) *VviNCED* expression as a function of elasticity. (**B**) The relationship between *VviNCED* expression and ABA concentration. (**C**) The relationship between sugar and ABA concentrations. Box treatment data include both boxed and unboxed berries because treatment had no significant effect. Each point represents the level of metabolite or expression in the skin or flesh of a single berry. The colour of the berries comprising each pool is shown.

In general, *VviNCED* expression was extremely low as E decreased from 10 to 2MPa (Fig. 7A). Although there were a few berries at high E that exhibited higher levels of expression, these levels were still <15% of peak levels. Large increases in *VviNCED* did not occur until after berries had changed colour (Fig. 7A). *VviNCED* expression was nearly undetectable as ABA concentration increased and there were no large increases in *VviNCED* until ABA concentration reached at least 4000ng g dry weight^−1^ (Fig. 7B). In green berries prior to colour development, ABA accumulated prior to sharp increases in sugars (Fig. 7C). ABA concentration reached near peak levels in green berries while sugar concentration continued to increase.

In Control berry skins both *VviMybA* and *VviUFGT* expression were undetectable in green berries during softening (Supplementary Fig. S5A). In Box berries the same was generally true, and after the boxes were removed expression levels increased. Sugar concentrations in Control berries increased significantly prior to increases in *VviMybA* and *VviUFGT* expression (Supplementary Fig. S5B). Sugar accumulation was largely eliminated in Box berries, but when the boxes were removed, sugars increased coincident with colour development (Supplementary Fig. S5B). *VviMybA* and *VviUFGT* expression were up-regulated in Box berries at much lower sugar concentrations than in Control berries. In relation to ABA, *VviMybA* and *VviUFGT* were not up-regulated until ABA reached high concentrations and the relationships between *VviMybA* and *VviUFGT* expression and increases in ABA concentration were nearly identical (Supplementary Fig. S5C).

## Discussion

These data demonstrate that softening and decreases in turgor along with increases in ABA represent some of the earliest events during the onset of ripening in grape. The initial ~80% of E decrease occurred with no increase in the expression of key cell wall-modifying genes or sugar concentration. Most of the major changes representing the onset of ripening occurred sharply after the berries reached low (< 2MPa) E, suggesting that this is a turning point in berry development. Increases in ABA occurred earlier during softening and prior to sharp increases in sugars and anthocyanins. ABA increased in the absence of significant expression of the rate-limiting *VviNCED*s and coincident with decreases in the predominant ABA catabolite diphasic acid, suggesting that the initial ABA increase is caused by decreased catabolism and/or exogenous import.

### Softening, decreases in turgor, and the onset of ripening

Despite the high variability in berry softening and berry size in the field, this study demonstrates that when observed across a large population of berries the majority of softening occurs prior to the resumption of growth and change in colour. Other studies that adopted E as a method of quantifying softening observed similar timing, showing that in several grapevine varieties P and E both start to decrease around 10 days before the transition from stage II to stage III (Thomas *et al.*, 2008; Wada *et al.*, 2008; Matthews *et al.*, 2009).

Two compatible mechanisms have been proposed for fruit softening: cell wall modification (Brummell and Harpster, 2001; Vicente *et al.*, 2007; Gapper *et al.*, 2013) and decreases in P (Shackel *et al.*, 1991; Tong *et al.*, 1999; Saladié *et al.*, 2007; Thomas *et al.*, 2008; Wada *et al.*, 2009; Matthews *et al.*, 2009). During stage II, when most of the softening occurred, our data illustrate a strong correlation between decreases in E and P consistent with previous reports (Thomas *et al.*, 2008; Wada *et al.*, 2009; Matthews *et al.*, 2009). Boxing experiments further strengthen the connection, demonstrating that boxing temporarily delayed decreases in both E and P.

This study investigated the expression of genes encoding five predominant cell wall modification enzymes. The genes were selected based on their previous characterization in relation to berry softening and the onset of ripening (Nunan *et al.*, 2001; Schlosser *et al.*, 2008). No significant expression of these genes was observed before E < 2MPa, demonstrating that the up-regulation of these key cell wall-loosening enzymes did not correspond with decreases in P and E, but did occur with the resumption of growth. Only two of the genes investigated were expressed before this time, *VviExp1* and *VviPME*, but at very low levels and only in flesh tissues. Similar results were found in grape by Nunan *et al.* (2001). In addition, the results presented here have striking similarity with those of Schlosser *et al.* (2008), who found that most cell wall-loosening enzymes were not significantly up-regulated until the sampling date at veraison (i.e. after decreases in P and E), with the exception of *VviPME*.

It is important to point out that this analysis is limited in two ways. The first is that the expression of a limited number of cell wall-modifying genes was examined, yet these genes were chosen because previous studies demonstrated they were the genes most highly expressed in grape berries. The second is that enzyme activity was not assessed. Good relationships between mRNA abundance of cell wall-related genes and compositional changes in the cell walls have been observed during fruit development and ripening (Nunan *et al.*, 2001). However, the activity of cell wall-modifying enzymes is under complex regulation (e.g. Reca *et al.*, 2012) and there remains the possibility that changes in cell wall composition do contribute to the early decreases in E.

Although the evidence for causative links between many of these cell wall modification enzymes and fruit softening *per se* is equivocal, many studies have shown that manipulating their activity in ripening fruits results in substantial changes in fruit texture and postharvest quality during the later stages of ripening (Brummell *et al.*, 2002; Powell *et al.*, 2003). This is consistent with the further up-regulation of these genes after colour development in the current study.

Whether there is a mechanistic relationship between turgor changes and the other ripening processes is still unknown. Our results demonstrated that changes in P and E occur earlier than increases in sugars and preceded the up-regulation of a host of ripening-related genes. This indicates that the triggering of ripening most likely involves processes that occur much earlier than previously thought, perhaps even during stage I when E values first begin to decline.

### Preventing growth

Preventing growth delayed decreases in E and P, maintaining higher levels of P by preventing cell expansion and, possibly, by reducing transpiration and hence water loss from the berry. Matthews *et al.* (2009) also prevented growth by boxing berries with perforated boxes (i.e. allowing some transpiration) and observed an intermediate effect where a portion of berries still underwent colour development. An actively transpiring berry would tend to have a more negative water potential than a non-transpiring berry, favouring the influx of water along with potential signalling compounds into the berry; a process that may be inhibited by boxing berries. These results suggest that berry expansion and the resulting changes in water relations are involved in the regulation of the accumulation of sugars and anthocyanins at the onset of ripening.

When compared to E in boxed berries, P remained higher until 3 days after the box was removed. This most likely reflects the fact that P can be measured without removing the berry from the box while measuring E requires removing it. Matthews *et al.* (2009) demonstrated a drop in P upon the removal of the boxes, thus E may behave similarly, being sustained at higher levels by the box. The necessary release of the berry from the box for the measurement likely resulted in slightly lower values of E than the real values. Nevertheless, the relationship between E and P in individual berries was extremely strong (Fig. 2C).

Preventing growth inhibited sugar accumulation and colour development. This result is consistent with previous studies in our laboratory where the disruption of phloem transport to berry clusters (via girdling) prior to the onset of ripening completely abolished colour development (unpublished data). Taken together, these data suggest that decreases in E and P are necessary but not sufficient for colour development. Furthermore, the data suggest that in grape berries anthocyanin biosynthesis requires increases in sugar concentration. This follows from numerous previous works demonstrating a regulatory role for sugar in stimulating anthocyanin biosynthesis in cultured grape tissues and berries, grape cell culture, and model systems (e.g. Larronde *et al.*, 1998; Loreti *et al.*, 2008; Gambetta *et al.*, 2010; Dai *et al.*, 2014).

### Softening and sugar accumulation

Berry Ψ_s_ and sugar concentration only changed significantly at E levels below 2MPa. This observation contrasts with Coombe (1992), who reported that changes in softening, which occurred 6 days before colour development, were associated with increases in hexose concentrations. This inconsistency likely results from the two studies’ different methodologies in quantifying softening. E is more sensitive to the initial changes in fruit firmness (E from ~10 to ~5 MPa) than the methodology used by Coombe and colleagues (Coombe and Bishop, 1980; Coombe and Phillips, 1982; Coombe, 1992). Thomas *et al.* (2008) showed that when firmness was recalculated as E for the data presented in Coombe (1992), the majority of fruit softening also occurred prior to rapid sugar accumulation.

The up-regulation of sugar-related genes was consistent with the increase in sugar accumulation. The major sugar-related genes involved in fruit development and ripening in grapevine are cell wall invertases, vacuolar invertases, and some hexose transporters (Davies and Robinson, 1996; Zhang *et al.*, 2006; Hayes *et al.*, 2007). Our data show that *VviInv* has its maximum expression in the flesh at an E of ~8–6MPa, in berries that are not accumulating sugars. Similarly, Hayes *et al.* (2007) reported that *VviInv* expression peaked just before ripening and was low during fruit ripening. Zhang *et al.* (2006) showed that cell wall invertase activity increases just before ripening and remains high during ripening.

In this study, there was an increase in the expression of hexose transporter genes after the onset of ripening in control berries. The expression of hexose transporters is mainly associated with sink tissues that import sugars from the apoplast (Agasse *et al.*, 2009). In grape berry *VviHT1*, *VviHT2*, and *VviHT3* are the most highly expressed compared to other *VviHTs* (Agasse *et al.*, 2009). However, other more recently identified hexose transporters such as tonoplast hexose transporter homologues (e.g. *VviHT6*) were not included in this study and might play critical roles in sugar accumulation (Lecourieux *et al.*, 2014).

### ABA metabolism, softening, and turgor

Previous studies identified peak ABA accumulation in grape berries at the onset of ripening and the ABA concentrations detected in this study were similar in magnitude (Davies *et al.*, 1997; Owen *et al.*, 2009; Deluc *et al.*, 2009). However, there are substantial methodological differences between this and previous studies. This study is the first to sample individual berries using a quantitative measure of firmness (i.e. elasticity, E) that controls for the substantial heterogeneity present between individual berries. Davies *et al.* (1997) and Deluc *et al.* (2009) carried out their analyses on pooled whole berries, while Owen *et al.* (2009) analysed pooled skins and flesh separately but at only two time points (the onset of ripening and approximately 2 months later).

In our study, berry ABA levels began to increase prior to the up-regulation of endogenous ABA biosynthesis, represented by the expression of the rate-limiting *VviNCED*s, but coincident with decreases in the predominant ABA catabolite DPA. This suggests that the initial increases in ABA may largely result from decreased catabolism; specifically decreases in 8′ hydroxylation. After subsequent increases in catabolism, ABA levels would be maintained via increases in the expression of the *VviNCED*s. Additionally, our results are consistent with a possible role of exogenous import. Shiozaki *et al.* (1999) found that ABA was immuno-localized in berry phloem tissues. The transport of ABA in phloem is common across a variety of plant species (Zeevaart, 1977; Hoad, 1978; Weiler and Ziegler, 1981) and is consistent with a shift in the predominant pathway of water transport into the berry from xylem to phloem at the onset of ripening (Greenspan *et al.*, 1994; Keller *et al.*, 2015).

ABA concentrations reached near peak levels prior to the up-regulation of the *VviNCED*s and colour development. The increase in ABA concentration is consistent with previous studies demonstrating the up-regulation of the *VviNCED*s by ABA (Wheeler *et al.*, 2009; Koyama *et al.*, 2010; Sun *et al.*, 2010). However, in the current study, increases in *VviNCED* expression did not correlate with significant increases in ABA concentrations. Instead, a sharp increase in *VviNCED* expression coincided with a marked increase in the concentrations of ABA-GE and ABA catabolites, suggesting that further increases in ABA are limited through a combination of conjugation and catabolism.

The data presented here show that decreases in P and E coincided with increases in ABA yet preceded increases in sugar concentration. Previous work in our laboratory demonstrated that supplying exogenous ABA and sucrose to pre-veraison grape berries in culture resulted in berry softening (Gambetta *et al.*, 2010), and subsequent studies have shown that ABA alone promotes berry softening (unpublished data). Interestingly, Box berries displayed similar levels of ABA to Control berries at 69 DAA, before the boxes were removed. This observation, together with the lack of sugar and anthocyanins in Box berries at the same stage, suggests that ABA by itself is not sufficient for sugar accumulation and colour development at the onset of berry ripening.

Although low turgor is classically thought to lead to increased ABA, there are at least two different mechanisms by which ABA could cause a decrease in turgor at the onset of ripening in grape. The first would be through the redistribution of solutes from the symplast to the apoplast. Wada *et al.* (2008) found that initial decreases in apoplast solute potential during softening are largely due to increases in the concentrations of malate and, to a lesser extent, sugars. The increase in extracellular malate provides striking parallels to ABA’s role in stomatal control, specifically the regulation of guard cell P, where extracellular malate plays a central role (reviewed in Kim *et al.*, 2010). In addition, ABA has been shown to stimulate the apoplastic accumulation of malate, albeit in a different developmental context (Heimovaara-Dijkstra *et al.*, 1994).

The second possible mechanism involves ABA and sugar. Zhang *et al.* (2006) found that at the onset of ripening phloem unloading shifted to the apoplast and the expression and activity of cell wall invertases increased, although the exact timing of the shift in phloem unloading relative to decreases in P was not determined. ABA has been shown to stimulate cell wall invertase activity in grape and other fruits (Pan *et al.*, 2005; Koyama *et al.*, 2010). This would result in decreases in P owing to both the accumulation of sugars in the apoplast and the cleavage of sucrose into glucose and fructose. In this study, decreases in P preceded any increase in sugar concentration. The apoplast represents a very small percentage of the tissue volume, likely allowing a significant decrease in apoplastic solute potential from a small numbers of solutes when compared with the symplast. Therefore, a relatively small increase in solutes in the apoplast through apoplastic phloem unloading, an increase of invertase activity, and/or solute redistribution could be responsible for the observed P loss (Wada *et al.*, 2008, 2009).

### Timing of events leading to the onset of ripening

**Fig. 8.**
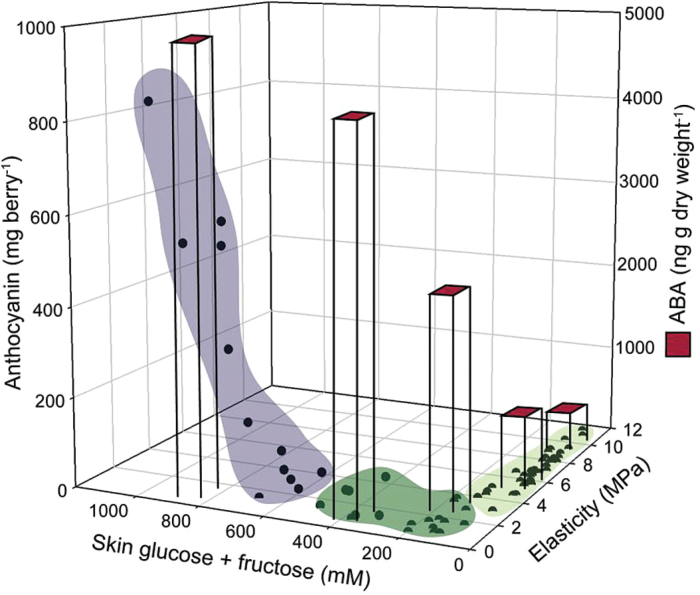
Summary of the relationship between decreases in elasticity (i.e. softening), sugar accumulation, increases in ABA, and anthocyanin accumulation (i.e. colour development). Points represent data for E, sugar concentration, and anthocyanin concentration measured in individual berries. Average ABA concentrations for selected elasticity pools are represented in the columns. Three sequential steps can be identified: 1 (light green) – E (and P) decreases, small increase in ABA concentration, no change in sugar concentration; 2 (dark green) – sugar concentration increases, large increase in ABA concentration, E (and P) decreases further; 3 (purple) – colour development begins.

The physiological changes, including softening, sugar accumulation, increases in ABA, and colour development, that occur at the onset of ripening were originally thought to be coincident. The data presented here delimit these changes into three phases (Fig. 8). The first two phases encompass the changes that occur as the berry softens in parallel with ABA accumulation (first phase) and sugar accumulation (second phase). Only after P and E have reached near minimum levels does anthocyanin accumulation and the third phase begin. This precise characterization of the timing of events during the onset of ripening is essential in understanding the ripening process. Furthermore, it can help guide future research on the regulation of distinct ripening events by focusing sampling within relevant periods and tissues.

## Supplementary material

Supplementary data are available at *JXB* online.

Table S1. Effect of the mock boxes on berry elasticity, diameter, weight, and colour.

Table S2. Grapevine V1 annotation codes of the genes analysed in the study.

Table S3. Evolution of solute potential (Ψ_s_) and glucose and fructose concentration in skin and flesh of Control berries across development.

Table S4. Evolution of solute potential (Ψ_s_) and glucose and fructose concentration in skin and flesh of Box berries across development.

Figure S1. Restricting growth of Zinfandel berries in the field.

Figure S2. Relationships between sugar accumulation and growth.

Figure S3. Relationship between anthocyanin accumulation and elasticity, and box effects on the percentage of coloured berries.

Figure S4. Individual berry elasticity in relation to DAA and growth.

Figure S5. Relationships between *VviMybA* and *VviUFGT* expression, sugars, and ABA in berry skins.

Supplementary Data
