# Supplementary material for: Characterization of major ripening events during softening in grape: turgor, sugar accumulation, abscisic acid metabolism, colour development, and their relationship with growth
Source: J Exp Bot. 2015 Nov 17;67(3):709–22. doi: 10.1093/jxb/erv483 (PMC4737070; doi:10.1093/jxb/erv483)
Supplement: Supplementary Data [file supp_67_3_709__index.html]

Characterization of major ripening events during softening in grape: turgor, sugar accumulation, abscisic acid metabolism, colour development, and their relationship with growth — Characterization of major ripening events during softening in grape: turgor, sugar accumulation, abscisic acid metabolism, colour development, and their relationship with growth — Supplementary Data 

# Characterization of major ripening events during softening in grape: turgor, sugar accumulation, abscisic acid metabolism, colour development, and their relationship with growth

## Supplementary Data

Data files

- Supplementary\_figures\_S1\_S5\_Tables\_S1\_S4.pdf - Supplementary Data
