# Supplementary material for: Characterization of major ripening events during softening in grape: turgor, sugar accumulation, abscisic acid metabolism, colour development, and their relationship with growth
Source: J Exp Bot. 2015 Nov 17;67(3):709–22. doi: 10.1093/jxb/erv483 (PMC4737070; doi:10.1093/jxb/erv483)
Supplement: Supplementary Data [file supp_erv483_Supplementary_figures_S1_S5_Tables_S1_S4.pdf]

Title: Characterization of major ripening events during softening in grape: turgor, sugar accumulation, ABA metabolism, color development, and their relationship with growth.

Authors: Simone D. Castellarin, Gregory A. Gambetta, Hiroshi Wada, Mark N. Krasnow, Grant R. Cramer, Enrico Peterlunger, Kenneth A. Shackel, Mark A. Matthews

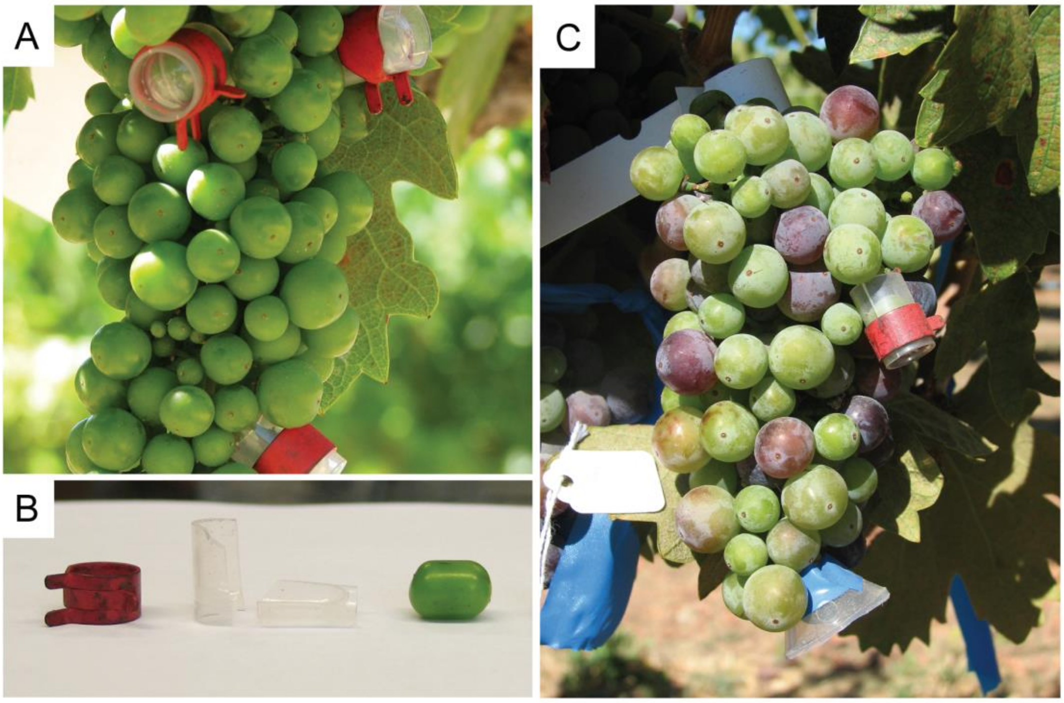

Figure S1. Restricting growth of Zinfandel berries in the field. A, Individual berries on whole clusters were boxed at random using modified cryotubes and tubing clamps. B, Boxes could be removed without harming the berry by simply removing the clamp. C, mock boxes were used as controls to assess possible indirect effects of on berry microclimate.

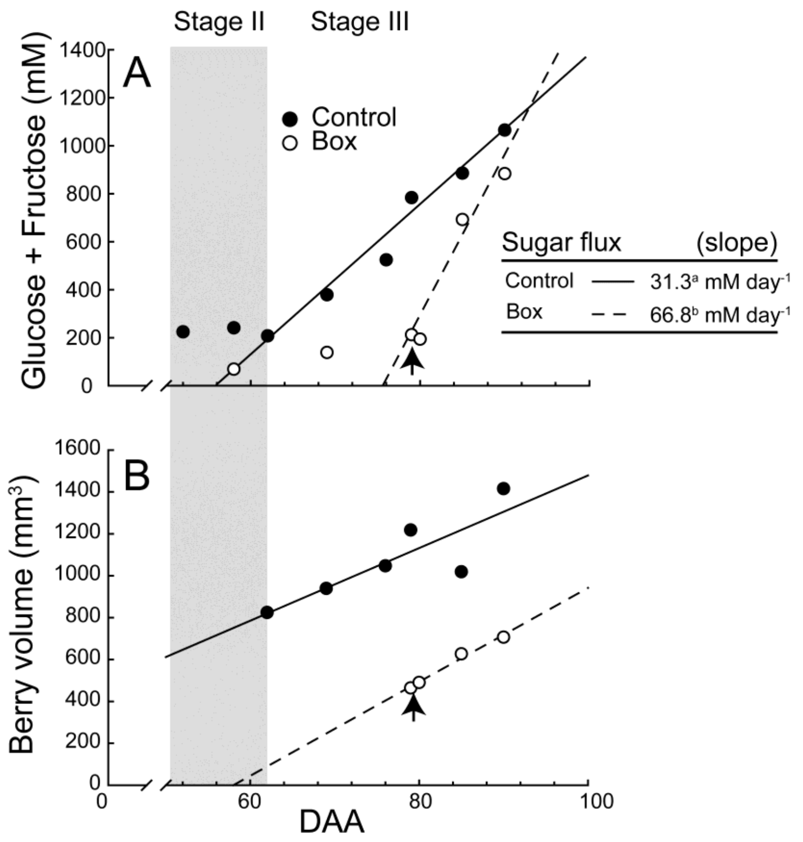

Figure S2. Relationships between sugar accumulation and growth in Control and Box. A, Rate of sugar accumulation (rate in Control and Box berries is reported in table). Slopes of linear regressions are significantly different (P = 0.004). Points are averages taken from Figure 4C. B, linear regressions of the relationship between berry volume (calculated from average berry radius data; Fig. 1) and DAA. Boxes were removed at 79 DAA (arrows).

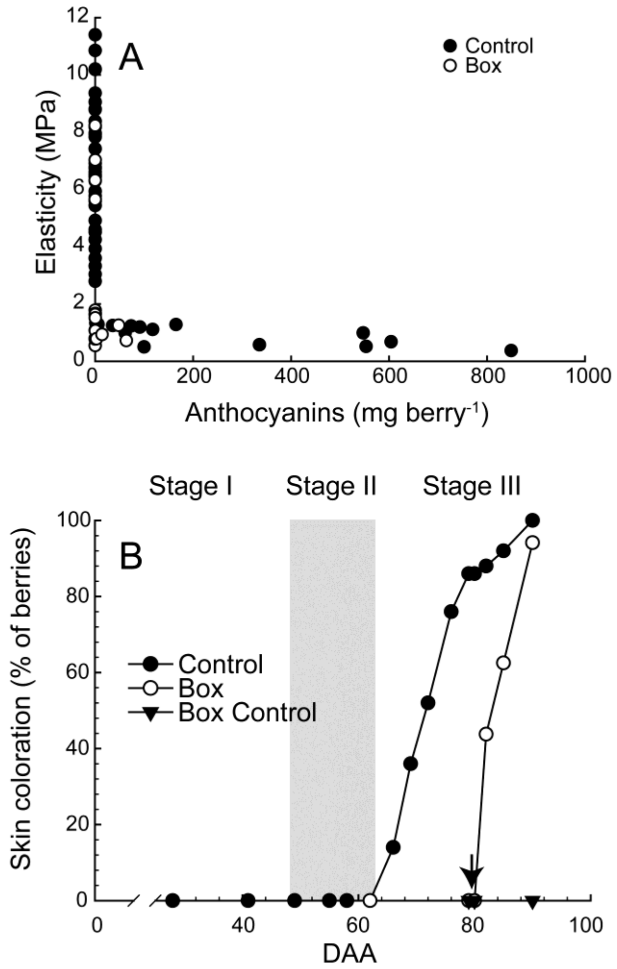

Figure S3. Relationship between anthocyanin accumulation and elasticity, and box effects on percent coloured berries. A, Anthocyanin accumulation in berry skins as a function of elasticity in Control and Box berries. B, Colour development in Control (n=50), Box, (n=12) and Box Control (n=1-3) berries. Boxes were removed at 79 DAA (arrows) and Box Controls remained boxed on the cluster and remained colourless for the duration of the experiment.

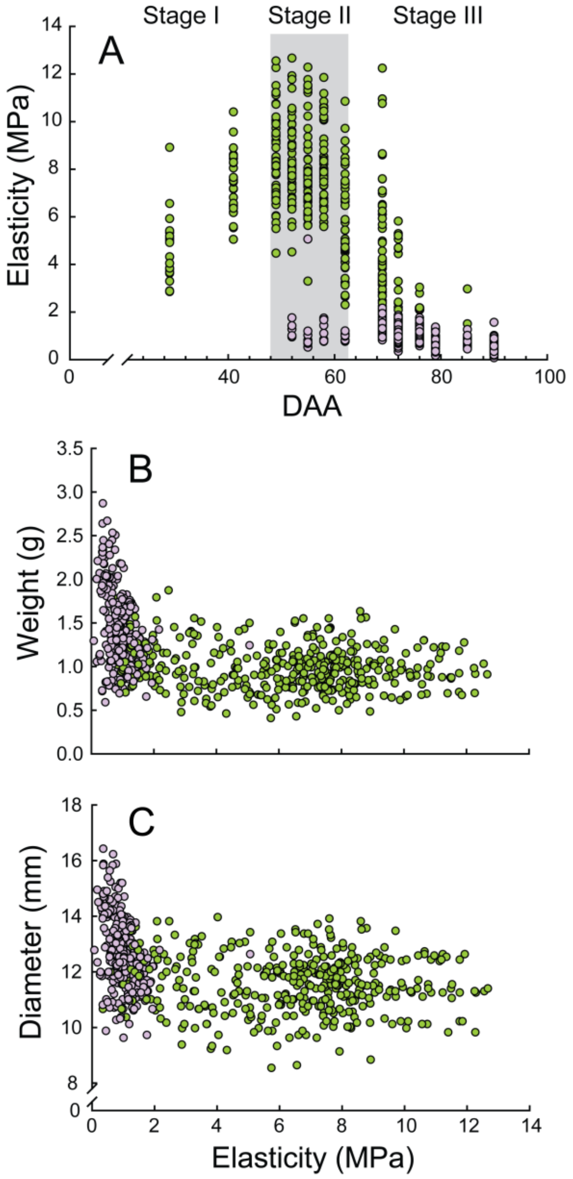

Figure S4. Individual berry elasticity in relation to DAA and growth. Values of 685 Control berries harvested from 29 DAA to 90 DAA are reported. Points are colored according to berry skin color. A, Development of fruit elasticity in the vineyard (n=20-100). B, Relationship between elasticity and berry weight. C, Relationship between elasticity and berry diameter.

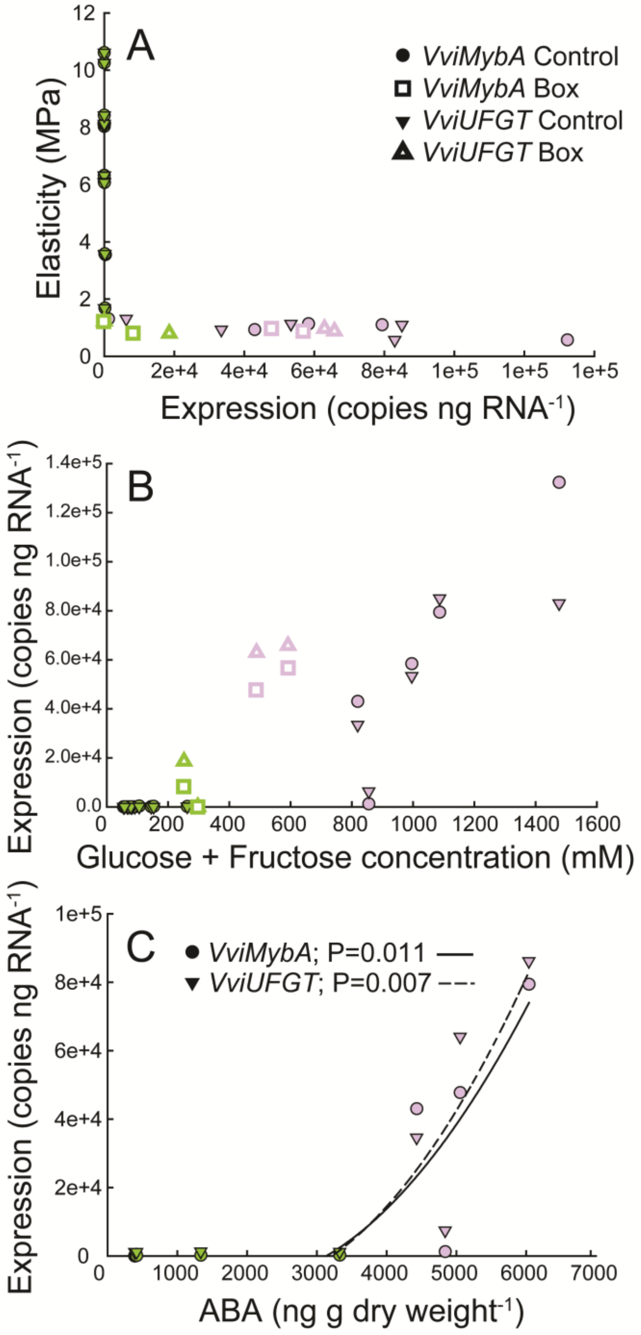

Figure S5. Relationships between *VviMybA* and *VviUFGT* expression, sugars, and ABA in berry skins. A, Expression of *VviMybA* (circles) and *VviUFGT* (triangles) in Control (filled) and Box (open). B, Relationship between *VviMybA* and *VviUFGT* expression and sugar accumulation. C, Relationship between ABA concentration and *VviMybA* and *VviUFGT* expression in control berries. Data presented is for berry skins only.

Table S1. Effect of the Mock boxes on berry Elasticity, diameter, weight, and color at 79 DAA (when the boxes were removed from Box berries). One way analysis of variance (n=12) was carried out and P values are reported. The different letters indicate significant differences (Tukey's HSD).

Treatment	Elasticity (MPa)	Berry diameter (mm)	Berry weight (g)	% of red berries
C	0.87 a	13.03 a	1.41 a	86
Mock	0.69 a	13.26 a	1.59 a	91
Box	1.06 a	9.60 b	0.98 b	0
P value	0.202	0.0001	0.0012	-

Table S2. Grapevine V1 annotation codes (Jaillon *et al.,* 2007) of the genes analyzed in this study. Primer sequences are reported in the related references.

Gene code	VIT code	Reference	Notes
VviUbi	VIT_16s0098g01190	Gambetta *et al.*, 2013	
VviExp1	VIT_18s0001g01130	Schlosser *et al.*, 2008	*VvEXPA19* in Del Santo *et al.*, 2013
VviExp2	VIT_13s0067g02930	Schlosser *et al.*, 2008	*VvEXPA14* in Del Santo *et al.*, 2013
VviPL	VIT_17s0000g09800<br>VIT_17s0000g09810	Schlosser *et al.*, 2008	
VviPME	VIT_12s0035g01900	Schlosser *et al.*, 2008	
VviXTH	VIT_06s0061g00550	Schlosser *et al.*, 2008	
VviHT1	VIT_00s0181g00010	Hayes *et al.*, 2007	
VviHT2	VIT_18s0001g05570	Hayes *et al.*, 2007	
VviHT3	VIT_11s0149g00050	Hayes *et al.*, 2007	
VviINV	VIT_09s0002g02320	Hayes *et al.*, 2007	
VviUFGT	VIT_16s0039g02230	Goto-Yamamoto *et al.*, 2002	
VviMybA	VIT_02s0033g00450<br>VIT_02s0033g00410<br>VIT_02s0033g00390<br>VIT_02s0033g00380	Castellarin *et al.*, 2007	
VviNCED1	VIT_19s0093g00550	Castellarin *et al.*, 2007	
VviNCED2	VIT_10s0003g03750	Castellarin *et al.*, 2007	

Table S3. Solute potential (Ψ_s), and glucose and fructose concentration in skin and flesh of control berries (Control) across development.

Controls	Ψ_s (MPa)			Glucose (mM)			Fructose (mM)		
DAA	Skin	Flesh	*P*	Skin	Flesh	*P*	Skin	Flesh	*P*
52	-0.89	-0.91	0.597	53.5	186.7	0.001	14.9	53.3	0.009
58	-0.92	-0.92	0.935	71.3	192.9	<0.001	15.4	62.8	<0.001
62	-0.96	-1.01	0.375	70.0	194.5	<0.001	6.6	66.0	0.007
69	-1.37	-1.29	0.714	234.8	230.1	0.946	177.6	142.0	0.623
76	-1.85	-1.75	0.839	316.9	310.7	0.963	196.5	214.1	0.858
79	-2.68	-2.33	0.452	541.4	457.0	0.466	291.3	322.9	0.705
85	-2.34	-2.67	0.592	449.5	533.6	0.567	237.9	372.8	0.246
90	-4.45	-3.37	0.277	724.6	691.0	0.739	494.1	360.0	0.284

Table S4. Solute potential (Ψ_s) and glucose and fructose concentration in skin and flesh of boxed berries (Box) across development.

Box	Ψ_s (MPa)			Glucose (mM)			Fructose (mM)		
DAA	Skin	Flesh	*P*	Skin	Flesh	*P*	Skin	Flesh	*P*
58	-0.78	-0.98	<0.001	62.9	67.0	0.796	18.2	nd	<0.001
69	-0.96	-1.10	0.479	72.3	106.2	0.559	46.5	34.0	0.815
79	-1.05	-1.09	0.796	114.3	141.6	0.503	97.1	69.3	0.460
80	-1.10	-1.17	0.450	136.5	130.4	0.861	109.9	57.7	0.156
85	-1.86	-1.85	0.988	245.6	417.6	0.104	167.0	300.0	0.255
90	-2.10	-2.20	0.822	308.4	1207.8	0.056	190.9	1127.1	0.059
